# Mango Fruit Yield and Critical Quality Parameters Respond to Foliar and Soil Applications of Zinc and Boron

**DOI:** 10.3390/plants7040097

**Published:** 2018-11-03

**Authors:** Iftikhar Ahmad, Fatma Bibi, Hameed Ullah, Tariq Muhammad Munir

**Affiliations:** 1 Department of Agriculture, Government of the Punjab, Mango Research Institute, Multan 60500, Pakistan; rocky_91294@yahoo.com (I.A.); fatima.bibi71@gmail.com (F.B.); hameedullah60@hotmail.com (H.U.); 2 Department of Geography, University of Calgary, 2500 University Dr. NW, Calgary, AB T2N 1N4, Canada; 3 Department of Geology, St. Mary’s University, 14500 Bannister Road SE, Calgary, AB T2X 1Z4, Canada

**Keywords:** aroma, flavour, fruit quality, fruit retention, fruit set, fruit yield, *Mangifera indica* L., nutrient, orchard, organoleptic, taste, texture

## Abstract

Mango (*Mangifera indica* L.), the sixth most important fruit crop worldwide, is likely at risk under a climate change scenario of accelerated soil organic matter mineralization and constrained plant nutrient supplies such as zinc (Zn) and boron (B). We identified the optimum nutrient formulation and application method to possibly rectify nutrient deficits in mango plants grown in one of the warmest and driest regions—Multan, Pakistan. We evaluated the yield and physiological (quality) responses of 20-year-old mango trees to seven treatments of foliar and soil applications of Zn and B. Combined soil application of B and Zn resulted in optimum increases in leaf mineral B and Zn and fruit-set, retention, yield, pulp recovery and total soluble solids at ripening (*p* = 0.021), while reducing titratable acidity and early fruit shedding (*p* = 0.034). Additionally, this treatment improved fruit quality (taste, flavour, texture, aroma, acceptability; *p* ≤ 0.05). Yield was found to be correlated with retention percentage (*P* ≤ 0.001; *R*^2^ = 0.91), which was in turn related to fruit-set number panicle^−1^ (*P* = 0.039; *R*^2^ = 0.61). Therefore, we suggest that combined soil application of B and Zn mitigates leaf mineral deficiencies and improves the yield and quality of mango more efficiently than other individual or combined foliar or soil treatments used in this study.

## 1. Introduction

Mango (*Mangifera indica* Linnaeus) is one of the most popular and earliest cultivated fruits of tropical and sub-tropical regions, and is grown in more than 100 countries [1]. It is ranked the second most cultivated tropical fruit and sixth major fruit crop worldwide [2], and the fruit has a high cropping potential under climate change scenarios [3]. Therefore, mango fruit appears to be one of the critical food sources for the consistently growing world population [2]. However, mango orchards face several problems like micronutrient deficiencies, physiological stresses, and fruit yield and quality challenges [4] that ultimately decrease production and exports. As far as we know, little attention has been paid to the optimum nutrient formulation that mitigates plant micronutrient deficiencies by foliar or soil application and improves fruit quality traits and yield.

Many of the mango-growing soils in Asia are calcareous, mostly intercropped, and received less than the optimum doses of fertilizers [1]. Additionally, soils are mainly deficient in zinc (Zn) and boron (B) e.g., [5]. Boron, an essential micronutrient, plays a critical role in the growth and enlargement of reproductive cells, initiation of flowering, and translocation of sugars [6]. Its deficiency is mostly transient and occurs during flowering and seed set. Prolonged lack results in premature shedding of flower or fruit, suggesting a higher demand for B during floral or fruit development [6]. Zinc is also known to be essential for metabolic processes and enzymatic and redox reactions occurring in plant cells. Zinc is directly involved in many plant growth processes, like the synthesis of specific amino acids [7]. Its deficiency is prevalent in mango orchards due to the calcareous nature of soils that do not support the micronutrient uptake. Most mango soils have imbalanced nutrient concentrations due to the exhaustive removal of nutrients by intercropped plants, augmented by partial or no soil replenishment, which results in long-term plant micronutrient deficiency.

Balanced application of fertilizers with Zn and B ensures optimum nutrient concentrations in leaves, which may lead to better quality and a sustainable increase in mango production. South Asian orchard soils are Zn- or B-deficient and may lead to reduced uptake of N and K by plants. Application of B and Zn improves the biochemistry of flowers and results in enhancing the fruit-set number per panicle and a fruit retention percentage for in achieving mango yield sustainability [5].

Low micronutrient use of Zn and B and poor management practices are mainly responsible for the reduction in yield and fruit quality in mango orchards, e.g., [8]. Uptake of soil-applied micronutrients is also reported to be low in mango orchards [9]. While soil application of the micronutrients in mango orchards is not extensively studied, foliar application of nutrients to pomegranate trees resulted in an increase in assimilation due to efficient mineral availability [10]. An individual or collective foliar application of Zn and B is also reported to improve fruit quantity, quality, pulp weight, total soluble solids (TSS) and ascorbic acid contents as compared to the control [1].

A field-based experiment was conducted in one of the densest mango-growing belts in Pakistan, using Chaunsa (white) cultivar, aiming to assess and understand how mango orchards respond to soil and foliar applications of Zn and B individually and combined. The central hypothesis was that collective soil application of Zn and B would increase fruit yield and improve fruit quality by mitigating the tissue micronutrient deficiencies. Therefore, specific objectives were to compare effects of foliar and soil-applied Zn and B on (1) leaf mineral Zn and B contents, (2) fruit retention, yield, and quality variables, over three growing seasons (2013–2015).

## 2. Results

The study orchard’s soil was loam in texture, low in organic matter, and had a pH of 8.42. The soil was deficient in available P, Zn, and B and adequate in K concentration (Table 1). No indications of salinity were found.

### 2.1. Yield and Quality Parameters

No significant between-year changes in fruit yield and quality traits were found; therefore, Table 2 shows only 3-y means. Application of foliar and soil B and Zn resulted in overall increases in fruit quality (pulp recovery, TSS, acidity) and yield (weight, volume) variables as compared to control (Table 2). The highest increases (*p* < 0.05) in fruit weight (320 g), volume (308 cm^3^), pulp recovery percentage (62%) and total soluble solids (17.8 brix), and a decrease in titratable acidity (0.32%) were recorded in response to T_5_. Similarly, T_7_ resulted in increases in fruit weight (274 g), volume (279 cm^3^), pulp recovery percentage (59%) and titratable acidity (0.35%).

### 2.2. Leaf Mineral Contents

The highest increase in leaf B and Zn contents occurred in the third year of the study in response to year-by-year applications of both nutrients (T_5_; *p* < 0.05; Table 3). Across all years, soil application of these micronutrients resulted in higher concentrations in leaves, as compared to control. T_5_ and T_7_ showed the highest Zn concentration in all years as compared to other treatments. Similarly, T_5_ and T_6_ were the highest in increasing leaf concentration of B as compared to other treatments in all years.

No significant changes in leaf mineral N, P and K were found between years; therefore Table 4 shows only 3-y means. Overall significant increases in leaf N, P and K contents were observed in response to Zn and B applications as compared to control. T_5_ resulted in the highest concentration of NPK (1.06%, 0.19%, 0.57%, respectively), compared to all other soil or foliar applications of micronutrients. Nevertheless, all micronutrient applications improved mineral contents in mango leaves in general.

### 2.3. Fruit Retention Percentage

**Figure 1:**
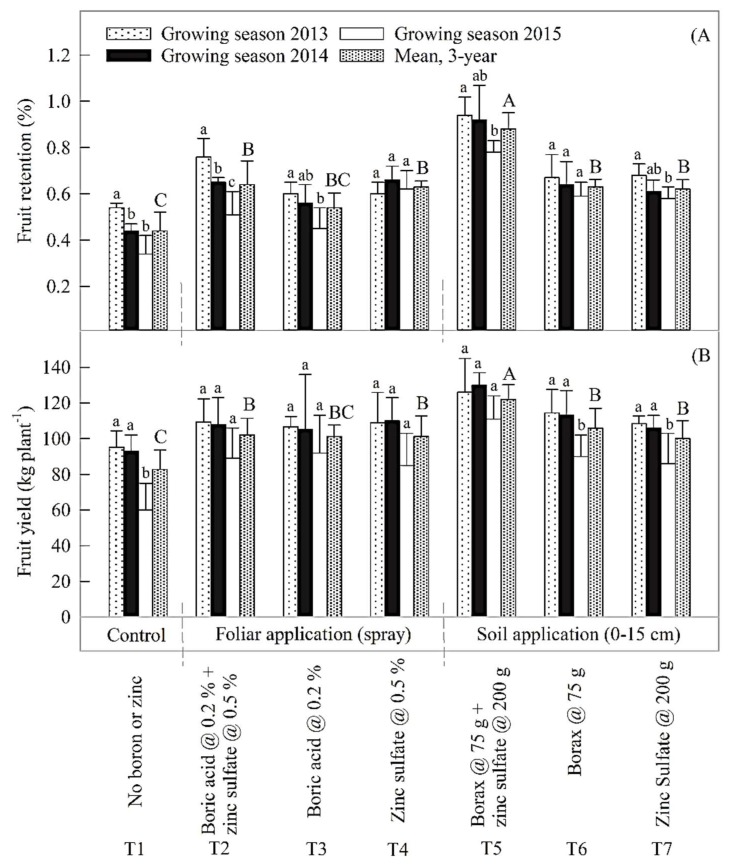
Effect of foliar and soil B and Zn treatments on: (**A**) mango fruit retention percentage and (**B**) fruit yield (kg plant^−1^). Between-year significant differences (*p* < 0.05) within a treatment are shown using lower case letters. Only 3-y means are being used to compare between-treatment significant differences (*p* < 0.05) shown using capital letters. Each error bar indicates ± SD of the mean (*n* = 4). Bars sharing the same letters are not different at *p* = 0.05.

We present responses of fruit retention and yield to foliar and soil B and Zn applications separately for all years as well as for 3-y means (Figure 1). A higher fruit shedding percentage (*p* < 0.05) and lower fruit yield (*p* < 0.05) were noticed in control as compared to those at all treated plots. The highest fruit retention percentage (0.88%) and fruit yield (118 kg plant^−1^) were found in response to T_5_ (*p* < 0.05). Second highest yield (106 kg plant^−1^) was observed in T_6_; it indicates that foliar application of Zn and B also resulted in yield increase but lesser than that in response to soil application. In T_5_, we observed an insignificant decrease in yield in the third year (86 kg plant^−1^). The third-year lesser yield could be due to corresponding lesser fruit retention percentage. In this treatment, during 2013 and 2014, the retention percentage was very high (0.94% and 0.92%) respectively, but in 2015 it remained insignificantly lesser than those in the last two years.

### 2.4. Correlations Between Fruit Yield and Quality Parameters

**Figure 2:**
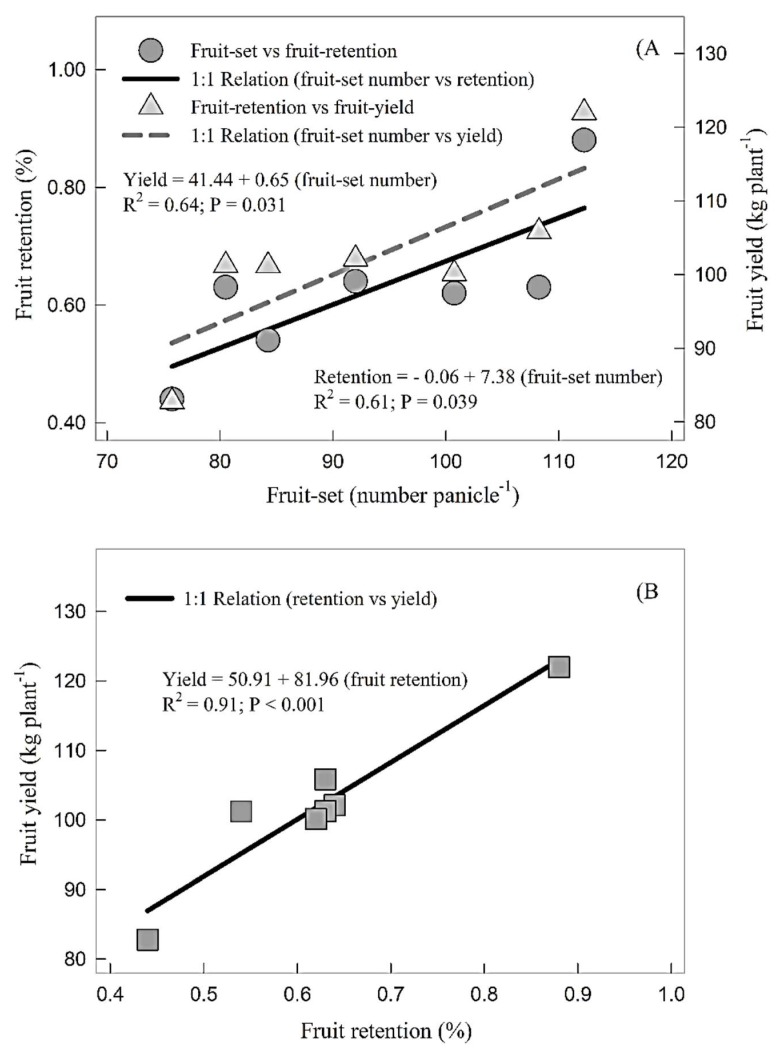
(**A**) Relationship between fruit-set number per panicle and fruit retention (%), and fruit yield (kg plant^−1^). Each point shows mean (*n* = 7) values. (**B**) Relationship between fruit retention percentage and fruit yield per plant. Each point shows means (*n* = 7) of fruit retention percentage, and fruit yield per plant. Every correlation showed significant at *p* < 0.05.

**Figure 3:**
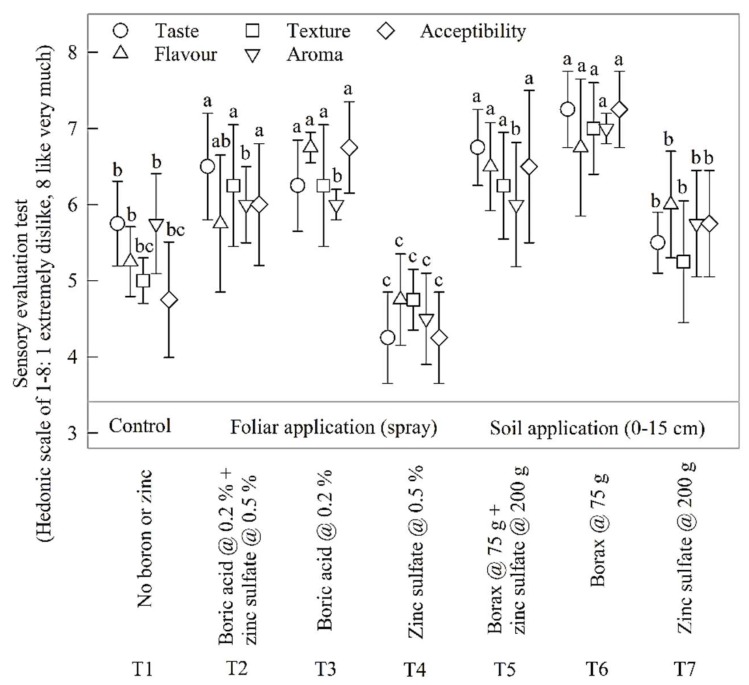
Spearman ranking correlation of five sensory evaluation fruit quality traits (*n* = 10): taste, flavour, texture, aroma and overall acceptability as affected by treatments. Within each treatment, all variables were correlated (*P* < 0.05). Each error bar indicates ± SD of the mean (*n* = 10). Bars sharing the same letters are not different at *p* = 0.05. Letters should be compared for each variable across treatments.

Fruit yield had significant and positive correlations with fruit-set number per panicle (*R*^2^ = 0.61; *P* = 0.039; Figure 2A) and fruit retention percentage (*R*^2^ = 0.91; *P* < 0.001; Figure 2B); fruit retention was in turn related with fruit-set number per panicle (*R*^2^ = 0.64; *P* = 0.031; Figure 2A). The highest leaf mineral B, Zn, N, P and K concentrations (Table 3 and Table 4), and the highest fruit-set number per panicle, fruit retention percentage and fruit yield (Figure 1) were recorded in response to combined soil application of B and Zn (T_5_). We also present the correlation between individual and clustered ranks assigned by 10 different testers based on sensory evaluation technique and Hedonic scale ranking (1–8) of five organoleptic traits: taste, flavour, texture, aroma, and acceptability (Figure 3). Overall, significant Spearman rank correlation (*R*^2^ = 0.68; *P* < 0.05 in all cases) was found between the rankings of mango fruit quality variables. Significant differences were also found between individual trait ranking among treatments, with the highest ranks in response to T_6_ and the lowest overall ranks to T_4_.

## 3. Discussion

This study found that the foliar and soil Zn and B applications improved the yield (fruit weight and volume) and quality (pulp recovery, TSS, Acidity) parameters of fruits in a mango orchard grown on normal loam soil in an arid region. Hasani et al. [11] reported similar findings of enhanced yield and better fruit quality (TSS, acidity, aroma, flesh colour and taste) in response to soil application of Zn as compared to control. In contrast, Masroor et al., (2016) [1] reported similar feedback from two foliar applications of Zn in November and March in comparison to our single foliar application at flowering. Supportive responses of yield and few quality variables of pomegranate fruits in response to soil applications were reported by Khorsandi et al. [12]. The increase of mango fruit quality and yield in response to the combined application of Zn and B may be due to improvements in sugar concentrations, vitamins and some physiological features [7].

The Zn foliar spray increased the leaf mineral content in sweet orange and Feutrell’s early mandarin plants [13]. They found that the soil application of borax was significantly better in increasing B availability to plant as compared to foliar spray as the sodium in the borax structure may cause leaf injury. Zia et al. [14] also concluded that the soil application of micronutrients was a better approach than foliar application in correcting deficiency impacts on quality and improving overall fruit yield. Due to the extensive occurrence of Zn and B deficiency in orchards it is, therefore, generally recommended applying both the micronutrients in soil e.g., [13]. However, to make up the deficiencies and consistently improve the yield and quality, foliar applications at the fruit-bearing stage is a significantly better practice [14]. Similar responses of purslane leaf mineral NPK concentrations to soil NPK applications were reported by Montoya-García et al. [15].

Optimum levels of leaf mineral contents improved fruit quality and crop yield (Table 3 and Table 4; Figure 1 and Figure 3) similar to the observations of dos-Santos et al. [16] and Antúnez-Ocampo et al. [17] in sunflower and ground cherry plants, respectively. They attributed the improved fruit quality and crop yield to a balanced use of Zn and B fertilizers. No antagonistic impacts of soil Zn and P were observed; though Razzaq et al. [18] found that P depressed the uptake of Zn when the two nutrients were applied in combination to the soil. Plant Zn content was found to be directly related to fruit drop as it is involved in the synthesis of tryptophan [19] or auxin (Indole Acetic Acid) [19,20] known for playing a critical role in fruit retention. Likewise, B application increased fruit-set number and yield; it could be due to improvement in reproductive development [21]. Deficiency of B is reported to increase fruit drop [9]. However, more research work is recommended to formulate nutrition component of best management practices for sustainable improvements in fruit quality and yield to be able to increase exports and meet food security challenges.

The fruit yield dependence on the number of fruit set per panicle and fruit retention percentage (Figure 2A) support the findings of our research as the fruit retention percentage was in turn dependent on the number of fruit set per panicle (Figure 2B). Moreover, the highest leaf mineral B, Zn, N, P and K concentrations (Table 3 and Table 4), and the highest number of fruit-set per panicle, fruit retention percentage and fruit yield (Figure 1) were recorded in response to the combined soil application of B and Zn in T5. More and thorough investigations including all possible combinations of foliar and soil applications need to be conducted to formulate best management practices for enhanced yields of better or export quality.

The sensory evaluation data of organoleptic parameters were subjected to rank correlation (Figure 3). There was an overall positive, between-parameters rank correlation across all treatments. Significant differences were also found between individual parameter ranks among all treatments, with the highest ranks in response to soil B application and the lowest fruit quality traits in response to 0.5% zinc sulfate foliar application in comparison to control. It is quite interesting that the same foliar application (0.5% zinc sulfate) led to an increase in fruit retention and yield in comparison to control (Table 1).

The negative response of taste, flavour and aroma traits of tree-ripe fruits to Zn spray could be due to the fact that zinc sulfate spray rapidly enhances the aroma and other quality traits of mango fruit e.g., [22], therefore, in this experiment the 0.5% zinc sulfate foliar application might have resulted in excessive production and accelerated losses of volatile aromatic compounds, mainly ethylene butyrate, as explained by Pesis (1996) for other fruits [23]. Additionally, the quality traits of different fruit species increase or decrease with time after picking or natural abscission; for example, mango fruit is known to have the best quality traits after picking and storage for five days [24] in comparison to feijoa fruit, which loses its quality traits during storage after natural abscission [25]. Further research is recommended to compare the taste and aroma traits of mango fruits in response to various nutrient application methods and treatments better by using e-tongue and e-nose sensing systems, as reported by Baldwin et al. (2011) [26] and Wilson and Baietto 2009 [27] for other fruits.

### Conclusions

Combined soil application of B and Zn at pre-flowering stage mitigates leaf mineral deficits and improves fruit yield and quality of mango more efficiently than other treatments at the specific experimental conditions. At ripening, significant increases in mango yield variables of fruit-set number per panicle, retention percentage, weight, volume, pulp recovery, total soluble solids, and decreases in titratable acidity and early fruit shedding were recorded. Significant improvements in quality traits of taste, flavour, texture, aroma, and acceptability were also observed in response to this treatment and in relation to yield variables.

## 4. Materials and Methods

### 4.1. Study Area

This research was conducted in an experimental mango orchard (30°09′ N, 71°26′ E; 410 m a.s.l.) located at the Mango Research Institute, Multan, Pakistan, over three growing seasons (2013‒15). According to climate data obtained from a meteorological station installed 3 km to the southwest at Central Cotton Research Institute, Multan, the three-year mean annual minimum and maximum air temperatures were 10.3 °C and 35.8 °C, respectively, with a mean annual precipitation of 82 mm.

### 4.2. Experimental Design

**Figure 4:**
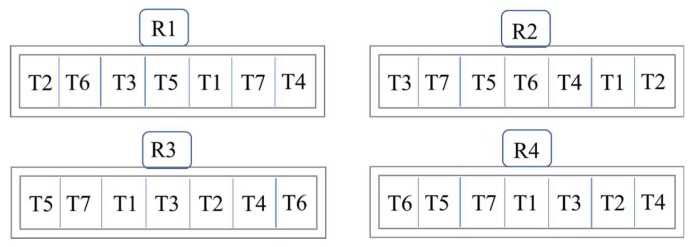
Graphical scheme showing the arrangement of seven treatments repeated four times in a randomized complete block design (RCBD) in the experimental mango orchard at Mango Research Station, Multan, Pakistan.

The experimental design was a randomized complete block, with seven treatments (T) and four replications (R) per treatment, and the experimental unit was a tree (Figure 4). Twenty-eight 20-year-old Chaunsa (cv. white) mango trees, equivalent in size and uniform in vigour, were selected in 2012 to study the effects of foliar and soil B and Zn applications on leaf mineral contents and fruit retention, yield, and quality traits. The trees were growing on soils with similar soil quality index [28] (data not provided) and were maintained using standard orchard management practices of irrigation, pruning, and weeding [29]. A balanced NPK basal dose (1000 g year^−1^) from urea (CO (NH_2_)_2_), single superphosphate (Ca (H_2_PO_4_)_2_ and potassium sulfate (K_2_SO_4_) sources, respectively, were applied to each tree: a full P (1000 g) and a one-half (500 g) each of the N and K mineral nutrients were mixed with topsoil under the canopy after fruit harvest (end of July 2012), and the remaining N and K were applied before flowering (start of February 2013). At the pre-flowering stage, foliar sprays or soil applications of Zn and B were also completed every year according to the following treatments and graphical scheme (Figure 4):

(T_1_) control; (T_2_) foliar spray: boric acid (H_3_BO_3_, 40 g @ 0.2%) + zinc sulfate (ZnSO_4_, 300 g @ 0.5%); (T_3_) foliar spray: boric acid (H_3_BO_3_, 40 g @ 0.2%); (T_4_) foliar spray: zinc sulfate (ZnSO_4_, 300 g @ 0.5%); (T_5_) soil application: borax (Na_2_B_4_O_7_.10H_2_O, 75 g) + zinc sulfate (ZnSO_4_, 200 g); (T_6_) soil application: borax (Na_2_B_4_O_7_.10H_2_O, 75 g); (T_7_) soil application: zinc sulfate (ZnSO_4_, 200 g).

### 4.3. Soil and Tissue Analyses

Three soil samples randomly taken from under each tree canopy were homogenized into a composite sample before the start of the experiment (June 2012) to determine the mean orchard soil characteristics. The pH and electrical conductivity (ECe) of soil were determined using saturated soil paste and soil extract methods, respectively. Available P and K were determined using Olsen’s P and flame photometric methods, respectively. The textural class was determined using the Bouyoucos method, while soil organic matter content was determined following Ryan et al. [30]. Zinc was evaluated by Diethylene Triamine Pentaacetic Acid extraction method using Atomic Absorption Spectrometer (AASPM—Shimadzu 7000, Kyoto, Japan), while B was first extracted with hydrochloric acid and then quantified by spectrophotometry. 

During each of the three growing seasons, 6–7-month-old leaves from the middle of non-fruiting shoots at the middle, top and bottom heights and all directions of the crown were sampled to determine leaf mineral N, P, K, Zn and B contents. The collected leaf samples were promptly transported to Microbiology Laboratory of the Mango Research Station, Multan. All leaf samples were thoroughly washed, rinsed with distilled water, oven dried at 70 °C in a convection oven until constant weight, ground in a Wiley’s Stainless-Steel Micro Mill (Swedesboro, NJ 08085, USA), passed through a 40-mesh screen and stored at room temperature in labelled plastic bags. We used the yellow-colour method with a triacid-digestion technique to determine the total P and flame photometric method to determine K. Aliquot was also used for B and Zn determinations using an Atomic Absorption Spectrometer (AASPM—Shimadzu 7000, Kyoto, Japan). The Kjeldahl distillation method was used for total N determination from plant tissue. 

### 4.4. Physiological and Yield Assessments

During each of the three fruit-set seasons, we quantified fruit retention percentage from a randomly selected/marked area of 1.0 m^2^ on each side of a randomly chosen tree in treatment on a monthly basis. At fruit harvest (end of July), marked areas were sampled to obtain an average fruit weight, and then the average yield (kg plant^−1^) was calculated by multiplying the average fruit weight with a total number of fruits. A top-loading, three decimal balance was used to measure fruit weight (g) after harvest (before and after ripening). Fruit volume (cm^3^) was measured using Archimedes’ principle (water displacement method) following Yan et al. [31].

### 4.5. Organoleptic and Chemical Assessments

Sensory evaluations of ripe mangoes were carried out by a panel of ten persons, technical staff of mango research institute in Multan. Taste, flavour, peel and flesh colour, texture, aroma and overall acceptability were tested using an eight-point hedonic scale. Titratable acidity of fresh mangoes for citric acid content (%) was measured using a standardized formula constructed by titrating sample juice (pH 8.2) with 0.1 N sodium hydroxide (NaOH) as described by Souza et al. [32]. The total soluble solids (TSS) in fresh mango juice were measured by using a digital hand refractometer (SELECT045, Medline Scientific Ltd., Chalgrove OX44 7XZ, UK).

### 4.6. Data Analyses

All data were statistically analysed using Statistix^®^ v 8.1 software (Tallahassee, FL 32317, USA). A repeated measure analysis of variance (ANOVA) was used to test the effects of foliar and soil B and Zn treatments on leaf mineral contents, fruit retention, fruit yield, and fruit quality variables. Since the same variables were quantified for 2013–15, the year was taken as fixed as well as a repeated measurement following Munir et al. [33]. Difference between treatment means was compared by Tukey’s significance difference test at *p* ≤ 0.05.

## Figures and Tables

**Table 1:** Soil physicochemical characteristics and mineral nutrient status of the study orchard *.

Soil Depth	Texture	Organic Matter	pH	ECe	P	K	Zn	B
(cm)	(class)	(%)		(dSm^−1^)	(mg kg^−1^)			
0–15	Loam	0.63	8.42	1.90	6.52	140	0.45	0.57
15–30	Loam	0.43	8.30	2.18	4.50	120	0.32	0.38

* Values are means (*n* = 28). Soil samples were collected before the start of the study in June 2012.

**Table 2:** Effect of experimental treatments on mean yield and quality traits of mango fruit (cv. Chaunsa white) *.

Treatment/Plant	Fruit Volume	Fruit Weight	Pulp Recovery	Total Soluble Solids	Titratable Acidity
	(cm^3^)	(g)	(%)	(brix)	(%)
(T1). Control	204 ^c^	214^c^	53 ^c^	14 ^c^	0.53 ^a^
(T2). Boric acid @ 40 g (0.2%) + ZnSO_4_ @ 300 g (0.5%)	240 ^bc^	230 ^c^	55 ^bc^	15 ^bc^	0.45 ^ab^
(T3). Boric acid @ 40 g (0.2%)	244 ^bc^	234 ^c^	56 ^bc^	16 ^abc^	0.37 ^bc^
(T4). ZnSO_4_ @ 300 g (0.5%)	254 ^bc^	240 ^c^	56 ^bc^	17 ^ab^	0.38 ^c^
(T5). Borax @ 75 g + ZnSO_4_ @ 200 g	308 ^a^	320 ^a^	62 ^a^	18 ^a^	0.32 ^c^
(T6). Borax @ 75 g	243 ^bc^	268 ^b^	57 ^b^	16 ^bc^	0.35 ^bc^
(T7). ZnSO_4_ @ 200 g	279 ^ab^	274 ^ab^	59 ^a^	16 ^abc^	0.35 ^bc^

* Values are means (*n* = 4) over three years. Means (in a column) are statistically different (*p* ≤ 0.05) if they have no letter in common.

**Table 3:** Effect of experimental treatments on mean B and Zn concentrations (mg kg^−1^) in mango leaves (cv. Chaunsa white) sampled in July (2013–2015) *.

Treatment/Plant	2013		2014		2015		3-Y Mean (*n* = 12)	
	B	Zn	B	Zn	B	Zn	B	Zn
(T1). Control	20.0 ^d^	19.5 ^c^	19.5 ^d^	14.5 ^c^	18.3 ^d^	16.0 ^c^	19.3 ^d^	16.7 ^d^
(T2). Boric acid @ 40 g (0.2%) + ZnSO_4_ @ 300 g (0.5%)	23.0 ^c^	23.8 ^ab^	24.0 ^bc^	22.8 ^ab^	23.0 ^c^	23.0 ^b^	23.3 ^cd^	23.2 ^bc^
(T3). Boric acid @ 40 g (0.2%)	24.3 ^bc^	20.3 ^c^	24.8 ^c^	19.0 ^b^	25.0 ^bc^	20.0 ^bc^	24.7 ^bc^	19.8 ^cd^
(T4). ZnSO_4_ @ 300 g (0.5%)	23.5 ^c^	24.3 ^a^	23.8 ^bc^	23.8 ^a^	24.0 ^bc^	24.0 ^b^	23.7 ^cd^	24.0 ^ab^
(T5). Borax @ 75 g + Zn SO_4_ @ 200 g	26.0 ^a^	25.5 ^a^	26.0 ^abc^	26.8 ^a^	29.0 ^a^	31.0 ^a^	27.0 ^a^	27.8 ^a^
(T6). Borax @ 75 g	25.5 ^ab^	21.5 ^bc^	27.0 ^a^	23.0 ^ab^	26.0 ^b^	21.0 ^b^	26.2 ^a^	21.8 ^cd^
(T7). Zn SO_4_ @ 200 g	24.5 ^bc^	25.3 ^a^	26.5 ^ab^	25.0 ^a^	26.0 ^b^	26.0 ^a^	25.7 ^bc^	25.4 ^a^

* Means (in a column) are statistically different (*p* ≤ 0.05) if they have no letter in common.

**Table 4:** Mean N, P, and K mineral contents in mango leaves (cv. Chaunsa white) sampled at the end of each crop year in July (2013–2015) *.

Treatment/Plant	Nitrogen	Phosphorus	Potassium
	(%)		
(T1). Control	0.85 ^d^	0.13 ^c^	0.49 ^b^
(T2). Boric acid @ 40 g (0.2%) + ZnSO_4_ @ 300 g (0.5%)	0.95 ^bcd^	0.13 ^c^	0.50 ^ab^
T3). Boric acid @ 40 g (0.2%)	0.90 ^cd^	0.14 ^bc^	0.45 ^b^
(T4). ZnSO_4_ @ 300 g (0.5%)	0.96 ^abc^	0.13 ^c^	0.52 ^ab^
(T5). Borax @ 75 g + Zn SO_4_ @ 200 g	1.06 ^a^	0.19 ^a^	0.57 ^a^
(T6). Borax @ 75 g	0.99 ^abc^	0.18 ^a^	0.48 ^b^
(T7). Zn SO_4_ @ 200 g	1.05 ^ab^	0.16 ^ab^	0.51 ^ab^

* Values are 3-y means (*n* = 4). Means sharing same letters do not differ at *p* = 0.05. Letters should be compared within a single column.
